# Editorial: Sustaining Innovation in Compassionate Free-Roaming Cat Management Across the Globe: A Decadal Reappraisal of the Practice and Promise of Trap-Neuter-Vaccinate-Return (TNVR)

**DOI:** 10.3389/fvets.2019.00365

**Published:** 2019-11-05

**Authors:** Joan E. Schaffner, Geoffrey Wandesforde-Smith, Peter Joseph Wolf, Julie Levy, Sophie Riley, Mark James Farnworth

**Affiliations:** ^1^The George Washington University Law School, Washington, DC, United States; ^2^Department of Political Science, University of California, Davis, Davis, CA, United States; ^3^Community Programs & Services, Best Friends Animal Society, Kanab, UT, United States; ^4^Maddie's Shelter Medicine Program, College of Veterinary Medicine, University of Florida, Gainesville, FL, United States; ^5^Faculty of Law, University of Technology Sydney, Ultimo, NSW, Australia; ^6^Animal, Rural and Environmental Sciences, Nottingham Trent University, Southwell, United Kingdom

**Keywords:** cats, feral cats community cats, trap-neuter-return (TNR), ethics, conservation, animal sheltering

In “A review of feral cat control,” published in the *Journal of Feline Medicine and Surgery* in 2008, Sheilah Robertson recognized that there was a clear, evolutionary trend in global thinking and advocacy about free-roaming cat management, moving away from lethal methods toward trap-neuter-vaccinate-return (TNVR). Although in some local circumstances the available data “support the success of TNR in reducing cat populations,” argued Robertson, “to have a large impact it will have to be adopted on a far greater scale than it is currently practiced” (1).

In the intervening period, advocacy of TNVR has remained strong particularly, but not exclusively, among local, national, and international animal welfare non-governmental organizations (NGOs), and in developed countries. However, there is some evidence that in countries where free-roaming cats are thought to pose a substantial threat to native species, lethal culling, and perhaps even the complete extermination, of free-roaming cats is being seriously contemplated as a matter of national policy (2, 3). Promoting such a policy appears to ignore the potential for effective TNVR activities and fails to account for public sentiment in favor of a more compassionate approach.

Ten years following the publication of Robertson's review, when this Research Topic was first conceived, we called on contributors representing a broad range of disciplines from across the globe to submit their latest research investigating various aspects of TNVR. Compiling the resulting articles, three distinct (but also, at times, overlapping) themes emerged: (1) human attitudes and beliefs regarding the management of free-roaming cats, (2) the effectiveness of TNVR as a management tool, and (3) the behavior and welfare of free-roaming cats.

## Human Attitudes and Beliefs Regarding Free-Roaming Cats

Employing structural equation models to analyze survey results from their case study from Bulwell, England, McDonald et al. developed a framework “whereby TNVR operations can be embedded within community engagement.” And successful engagement requires a deep understanding of a range of underlying factors: “the drivers of behavioral intention go far beyond a lack of awareness alone… attitudes, perceptions and knowledge are all significant drivers.” Wolf and Schaffner examine some of these same drivers through the lens of “our evolving ethics.” Focusing on an aspect of the issue that Robertson left largely untouched, the authors situate the current trend toward TNVR as a preferred management scheme within the larger sociocultural context, specifically the “profound shift away from an anthropocentric utilitarian ethical framework toward a zoocentric virtue-based ethical framework.”

In one of three articles from researchers in Australia, where the government has publicly “declared war” on feral cats (4), Riley provides a historical perspective on the “changing legal status of cats… from friend of the settlers, to enemy of the rabbit, and now a threat to biodiversity and biosecurity risk.” Although TNVR is “unlikely to provide a complete solution to the problem of free-roaming cats in Australia,” Riley argues for its inclusion in policymaker's “suite of official measures.” Also reporting from Australia, Rand et al. present the results of their survey of Brisbane residents. “After being informed about [TNVR] programs for management of urban stray cats,” explain the authors, 79% indicated a preference for TNVR while 18% agreed with the city's current practice of lethal control, with the remaining 3% choosing to “leave the cats alone.” In a related article, Rand et al. examined the “perceptions of support and opposition from various stakeholders” among individuals involved with TNVR in Australia through an online questionnaire. Their results highlight the potential conflicts faced by practitioners and “authorities, landowners, neighbors, and people living and working in the area,” prompting the authors to conclude that there is a “need for legislative change to facilitate best-practice TN[V]R.”

## The Effectiveness of TNVR as a Management Tool

Analyzing 23 years of cat census data and veterinary records for more than 2,500 cats (including 1,691 sterilization surgeries), Kreisler et al. documented a 55% decrease in the population of free-roaming cats in the Key Largo, Florida, community they studied, as well as improved welfare “as measured by increased average age of population and decreased retrovirus prevalence.” Natoli et al. provide an update to an often-cited 2006 article (5) with their examination of 30 years of data to investigate the impact of a series of Italian laws designed to protect free-roaming cats, the first of which was implemented in 1991. Since 1988, 1,878 colonies have been registered in Rome alone, 89 (4.7%) of which have been eliminated and another 204 (10.8%) of which are considered stable, as a result of ongoing TNVR efforts. Hamilton details the steps involved as Hillsborough County, Florida, adopted three related programs (spay/neuter vouchers; TNVR; and a shelter-based version of TNVR commonly known as return-to-field, or RTF) for reducing the number of cats entering its shelter system and increasing the number leaving alive. Over 12 years, feline intake decreased by 51% and the municipal shelter's live-release rate reached 81.8% in 2017. Spehar and Wolf document the results of six large-scale U.S. shelter-based programs that integrated return-to-field (for “strays” brought to the shelter) and targeted TNVR, finding median reductions of 32% in feline intake and 83% in feline euthanasia. In addition, the authors report that the 72,970 cats enrolled were generally in good health, with only 0.5% euthanized due to serious health concerns. And, building upon their previous research (6), Boone et al. used stochastic modeling to compare seven scenarios for managing free-roaming cats (e.g., low- and high-intensity removal, episodic culling, and TNVR). Their findings highlight the importance of intensity “not only to reduce populations more quickly, but also to minimize the number of preventable deaths that occur over time.”

## The Behavior and Welfare of Free-Roaming Cats

As in Australia, free-roaming cats are an especially contentious issue in New Zealand. Using a newly developed “5-component visual health-related welfare assessment scale,” Zito et al. found no statistical differences between the apparent health and welfare of their samples of free-roaming pet cats, managed stray cats, and unmanaged stray cats in Auckland, providing “a starting point for further research that is urgently needed in this area.” Bruce et al. used small video cameras and global positioning system (GPS) technology mounted to break-away collars to document the activities of 37 free-roaming cats in Auckland, New Zealand. The authors report predation among 23 of the cats (62%) with 33% of events resulting is successful prey capture (46% invertebrates and 7% skinks; no mammals, birds, or amphibians). A total of 326 risk behaviors was observed among 32 cats, mostly cats “venturing onto the road.”

## Conclusion

Robertson's 2008 review (1) concludes with the observation that “the scientific literature on feral cats is increasing and is essential for modifying and improving current control methods.” As the articles compiled for this Research Topic illustrate, this body of literature has expanded considerably over the past decade, demonstrating TNVR's value as a tool for managing free-roaming cat populations.

## Author Contributions

PW drafted the editorial, which was reviewed and accepted by JS, GW-S, JL, SR, and MF.

### Conflict of Interest

The authors declare that the research was conducted in the absence of any commercial or financial relationships that could be construed as a potential conflict of interest.
